# Graph Neural Networks as a Potential Tool in Improving Virtual Screening Programs

**DOI:** 10.3389/fchem.2021.787194

**Published:** 2022-01-20

**Authors:** Luiz Anastacio Alves, Natiele Carla da Silva Ferreira, Victor Maricato, Anael Viana Pinto Alberto, Evellyn Araujo Dias, Nt Jose Aguiar Coelho

**Affiliations:** ^1^ Laboratory of Cellular Communication, Oswaldo Cruz Institute – Fiocruz, Rio de Janeiro, Brazil; ^2^ National Institute of Industrial Property - INPI and Veiga de Almeida University - UVA, Rio de Janeiro, Brazil

**Keywords:** GNN, deep learning, drug discovery, virtual screening, natural products

## Abstract

Despite the increasing number of pharmaceutical companies, university laboratories and funding, less than one percent of initially researched drugs enter the commercial market. In this context, virtual screening (VS) has gained much attention due to several advantages, including timesaving, reduced reagent and consumable costs and the performance of selective analyses regarding the affinity between test molecules and pharmacological targets. Currently, VS is based mainly on algorithms that apply physical and chemistry principles and quantum mechanics to estimate molecule affinities and conformations, among others. Nevertheless, VS has not reached the expected results concerning the improvement of market-approved drugs, comprising less than twenty drugs that have reached this goal to date. In this context, graph neural networks (GNN), a recent deep-learning subtype, may comprise a powerful tool to improve VS results concerning natural products that may be used both simultaneously with standard algorithms or isolated. This review discusses the pros and cons of GNN applied to VS and the future perspectives of this learnable algorithm, which may revolutionize drug discovery if certain obstacles concerning spatial coordinates and adequate datasets, among others, can be overcome.

## Introduction

Mathematic modeling comprises a valuable important tool in the development of the pharmacology and chemistry fields since their beginning as formal disciplines (Gaddum, 1953; Atkinson and Lalonde, 2007; Finlay et al., 2020). Thus, most traditional pharmacologists and chemistries are very accustomed in employing math modeling to solve or aid current issues regarding the development of new drugs.

**FIGURE 1:**
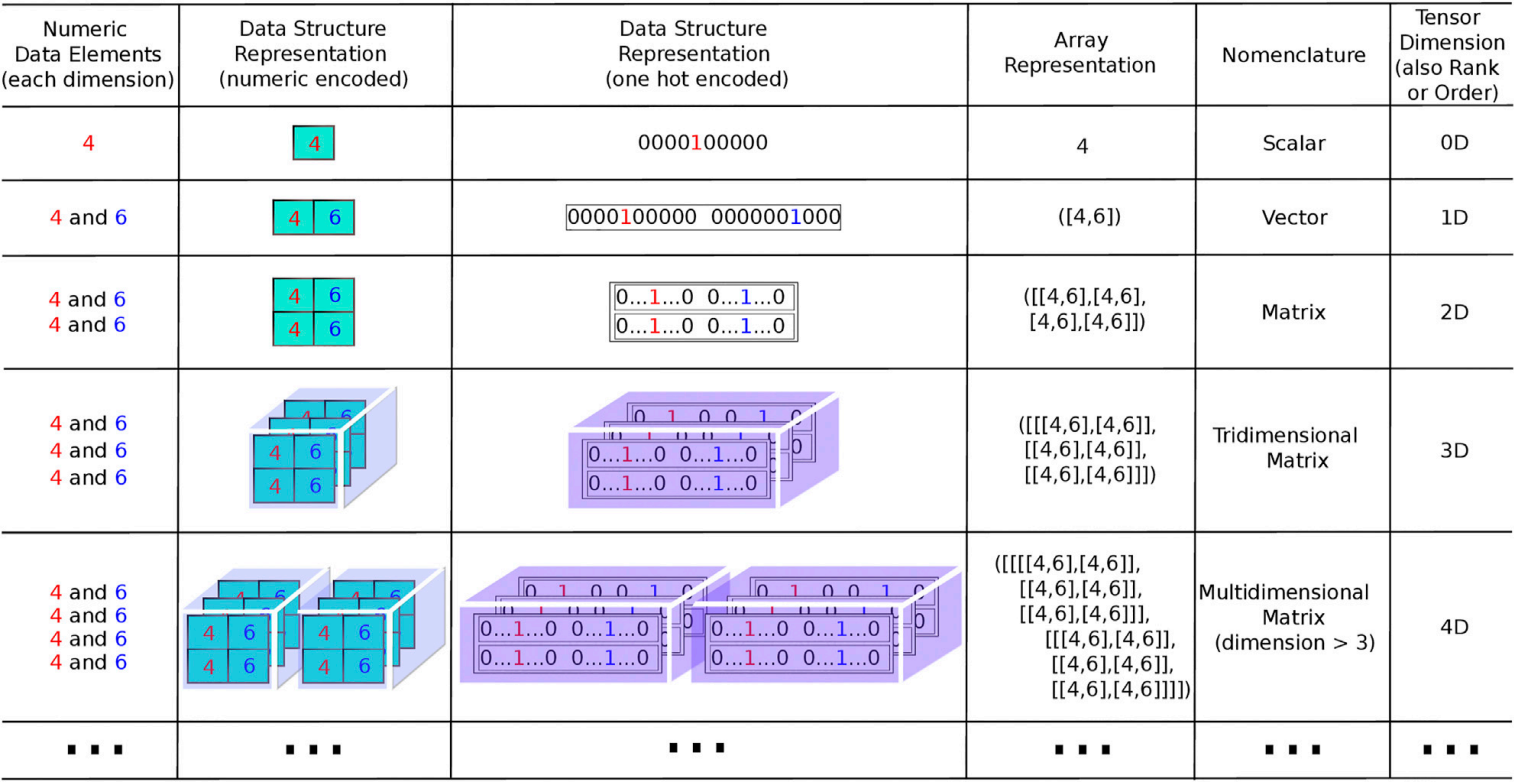
Representations of tensors in different orders (dimension, rank). Column 1 lists the elements that compose the sets corresponding to each dimension. The number of sets is in agreement with the number of dimensions except for the dimension 0, where one set is used as in dimension 1, but comprises a unitary set, as one element only is used to represent a scalar. More elements could have been used in each set for dimension 1 and above, but only two elements were used to reduce the complexity of the figure, comprising the same two elements in all dimensions. Column 2 contains elements “boxed” and spatially arranged in correspondence with the respective data structure. These comprise a row vector of “boxes” for dimension 1, a matrix with rows and columns of “boxes” for dimension 2, a block with rows, columns and pages for dimension 3, a row of blocks for dimension 4 and so on. Column 3 exhibits an equivalent arrangement as in the preceding column, except that each element is represented with “one-hot” encoding instead of numeric encoding. The “one-hot” representation is usual in neural networks to input information representing a set of features. Each feature is represented by a finite set of possible values of a specific feature attribute, with values ordered as positions in a sequence of values “0” and “1.” As an example, if a specific feature has an attribute summarized as having N possible values, it is represented by a sequence where one numeric value “1” is located in correspondence with the observed value of the attribute and N-1 values “0” are located in the positions corresponding to the other values. The input vector for a neural network is, thus, composed by a longer sequence chaining the “one-hot” representation of all features. Column 4 of the table presents examples of tensor representations as they are usually coded in computational language (Python coding, for example). At the beginning and ending of the data representation, the number of square brackets must conform to the tensor dimension. Columns 5 and 6 are the nomenclature of each kind of tensors up to rank 4 and its respective dimensions.

Computation power has increased enormously since the transistor invention, amplifying the use of diverse algorithms to aid in molecular modeling assessments. In the last decade, graphics processing unit (GPU) in parallel with math operations and tensor processing unit (TPU) applied to tensors comprise a very simplified definition of “multidimensional vectors” or for computer work “multidimensional arrays” (Figure 1), consolidating computational power. In addition, several free cloud computing platforms and web servers are now available to perform virtual screening (Gorgulla et al., 2020; Sigh, 2021). These developments have allowed for the development of the chemoinformatic field, that applies the knowledge of different disciplines, such chemistry, computation, math, physics, and biology to decipher chemical problems (Gasteiger, 2016, Chen et al., 2020). The first chemoinformatic assessment was reported by Ray and coworkers (1957), who employed a new algorithm to detect molecular substructures.

Recently, a new paradigm consisted in the use of artificial intelligence (AI), also named computational intelligence, as a chemoinformatic tool. No standard AI definition is noted in the literature (Dobrev, 2005; Kok et al., 2009), although one can assume characteristics linked human intelligence, as established in the Turing test (Kok et al., 2009). AI implementation is performed through of the application of machine learning (ML) algorithms. Several algorithms have now been applied to drug discovery, such as Random Forest, Support Vector Machine, K-Nearest Neighbors, Naïve Bayesian, Decision Trees, and Deep Learning, among others, and Publications from 2006 to 2016 indicate an increasing application of deep learning and other ML algorithms in drug discovery (Lavecchia, 2015; Jing et al., 2018; Maltarollo et al., 2019).

This review focus on a subtype of deep learning algorithm named graph neural network (GNN), currently one of the most applied. Despite being recent, the use of deep learning algorithms employing GNN may revolutionize the VS field, considered by some authors as the state of the art due to its high accuracy rates (Gaudelet et al., 2021). This article is mainly directed towards scientists, teachers and students that work with drug discovery or enthusiasts concerning this topic. Thus, a more didactic language and clear figures will be employed to clarify this theme and, perhaps, “recruit” new users of this technology.

### Virtual Screening

The concept of virtual screening first appeared in 1995 as a tool to test virtual libraries employing computers (Burns, 1995; Horvath, 1997). However, these ideas had begun in previous studies carried out in 1970s, such as Beddel and coworkers (1973), who worked on the spatial coordinates of hemoglobin modulators. At the time, several pharmaceutical companies were developing and applying screening projects to discover new antibiotics and drugs. Concurrently, cyclosporine, an immunosuppressive drug, was discovered through manual screening processes (Stahelin, 1996), later becoming the main drug used in organ transplants, saving millions of lives (Linden, 2009). In 1984, natural product automation was established, allowing for 10,000 assays a week (Pereira and Williams, 2007). Thus, began the high-throughput screening (HTS) age, that allows for the quick and efficient analysis of *in vitro* samples through bioassays that monitor the behavior of enzymes, secondary messengers, ion channels or biological effects associated with the investigated molecular target. These processes, however, are expensive and time consuming. In this context, virtual high-throughput screening (VHTS) is cheaper, employing computational facilities and diverse algorithms to detect the best ligands for a certain target that generally comprises proteins or nucleic acids. VHTS can be categorized into at least three types, namely ligand-based drug design (LBDD), structure-based drug design (SBDD), and hybrid methods [discussed in detail by Leelananda and Lindert (2016)].

Basically, LBDD methods are generally applied when the properties and ligand structure aspects are known, whereas the target structure (protein, receptor, ion channel and enzyme) is still unknown. LBDD may employ other methodologies, such as quantitative structure activity relationship (QSAR), and pharmacophore characteristics (or features) (Leelananda and Lindert, 2016). Techniques related to LBDD may also be used to perform toxicity studies by organic compounds named quantitative structure toxicity relationship (QSTR) (Jana et al., 2020). Structure-based drug design is mainly employed when the structure of target is known through the application of structural methodologies, such as X-ray diffraction, nuclear magnetic resonance and cryo-electron microscopy, or through computational methods, like homology modeling.

However, despite VS advances, less than twenty molecules are currently marketed in this regard (Kar and Roy, 2013; Leelananda and Lindert, 2016; Maia et al., 2020). In this context, the use of GNN may improve the numbers available drugs. In terms of natural products, at least 26 different types of virtual libraries are now available, in which thousands of molecules derived from natural products are registered. Some contain molecules from plants used in Traditional Chinese or African Medicine, such as the TCMID (The Traditional Chinese Medicine Integrated Database) and AfroDb (African Medicinal Plants Database), besides the Brazilian biodiversity database NUBBE (Nuclei of Bioassays, Ecophysiology and Biosynthesis of Natural Products Database) (Chen et al., 2017; Pilon et al., 2017).

### Deep Learning: Interdisciplinary Architecture

Several definitions for deep learning have been well discussed by Zhang et al., and all indicate an increase in feature hierarchy knowledge (Zhang et al., 2018). As Paulo Freire, the famous Brazilian Educator, said to state a new knowledge must be part of the learner’s life. In this context, deep learning is now a part of our lives in the form of the recommendation systems of several companies, social media (Facebook, Instagram, and Twitter, among others), the personal assistants of different operational systems, virtual bank assistants and self-driving cars, just to name a few. Currently, several deep learning implementations are noted in speech and image recognition systems and natural language processing (LeCun et al., 2015). The term “deep” in deep learning indicates a hidden layer or “hidden neurons,” as this network is initially based on neural functioning, and it is important to compare “math neurons” to biological neurons to better understand deep learning impacts in any modern world area.

**FIGURE 2:**
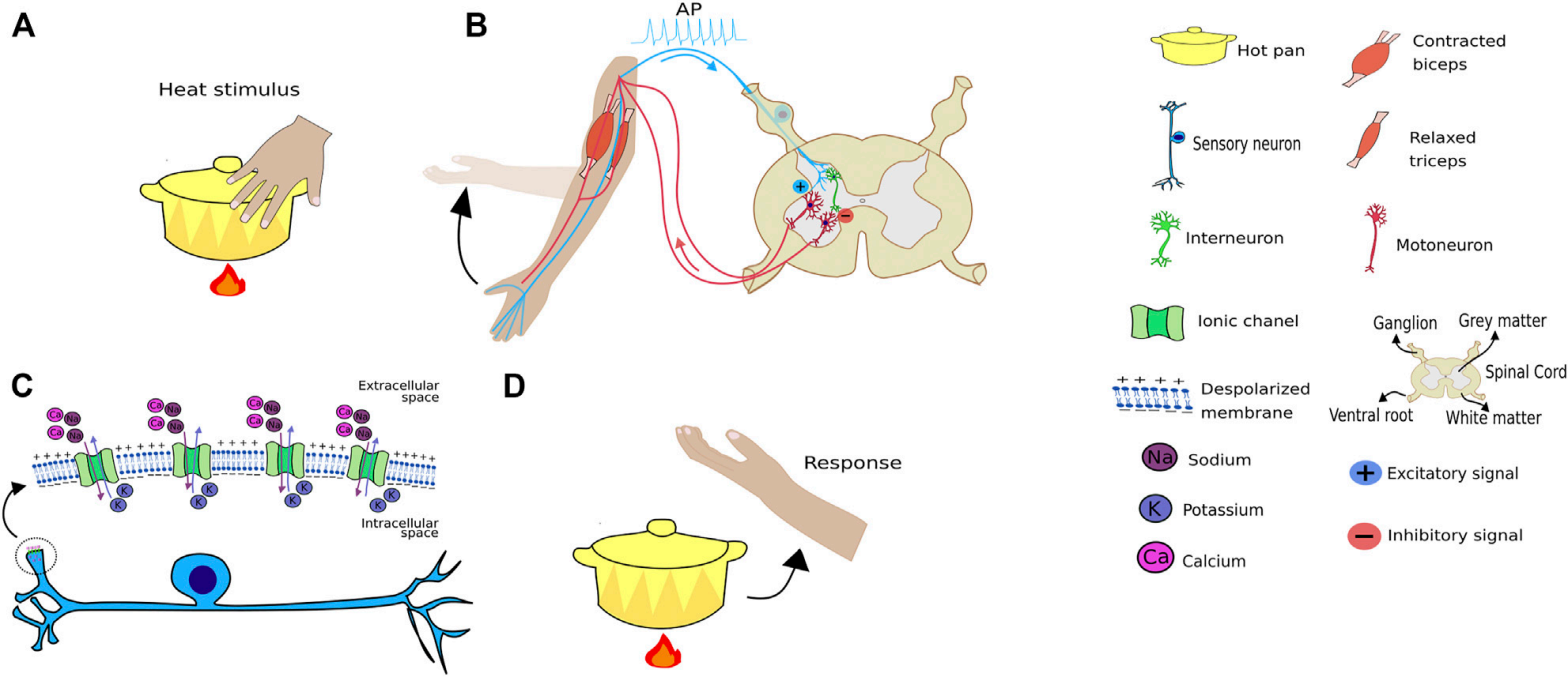
Heat stimulus transmission. **(A)** Schematic representation of a hand touching a hot pan and receiving a temperature sensory receptor stimulus. **(B)** The stimulus provokes a signal that will travel through a sensory neuron into the spinal cord, releasing excitatory neurotransmissions when it synapses with an alpha motoneuron that innervates the biceps. The sensory neuron will also synapse with an interneuron, that synapses with an alpha motoneuron that innervates the triceps, although in this case inhibitory neurotransmitters are released, causing the triceps to relax, thus, facilitating the withdraw reflex. **(C)** Channels from the TRP family expressed in sensory neurons will open following the thermal stimulus, leading to an entrance of cations such as sodium, resulting in membrane depolarization that could trigger an action potential. **(D)** Reflex of removing the hand from the heat after touching the pan. It is important to note this movement is performed through the association of inhibitory and excitatory synapses that work as positive and negative weights in artificial neural networks.

**FIGURE 3:**
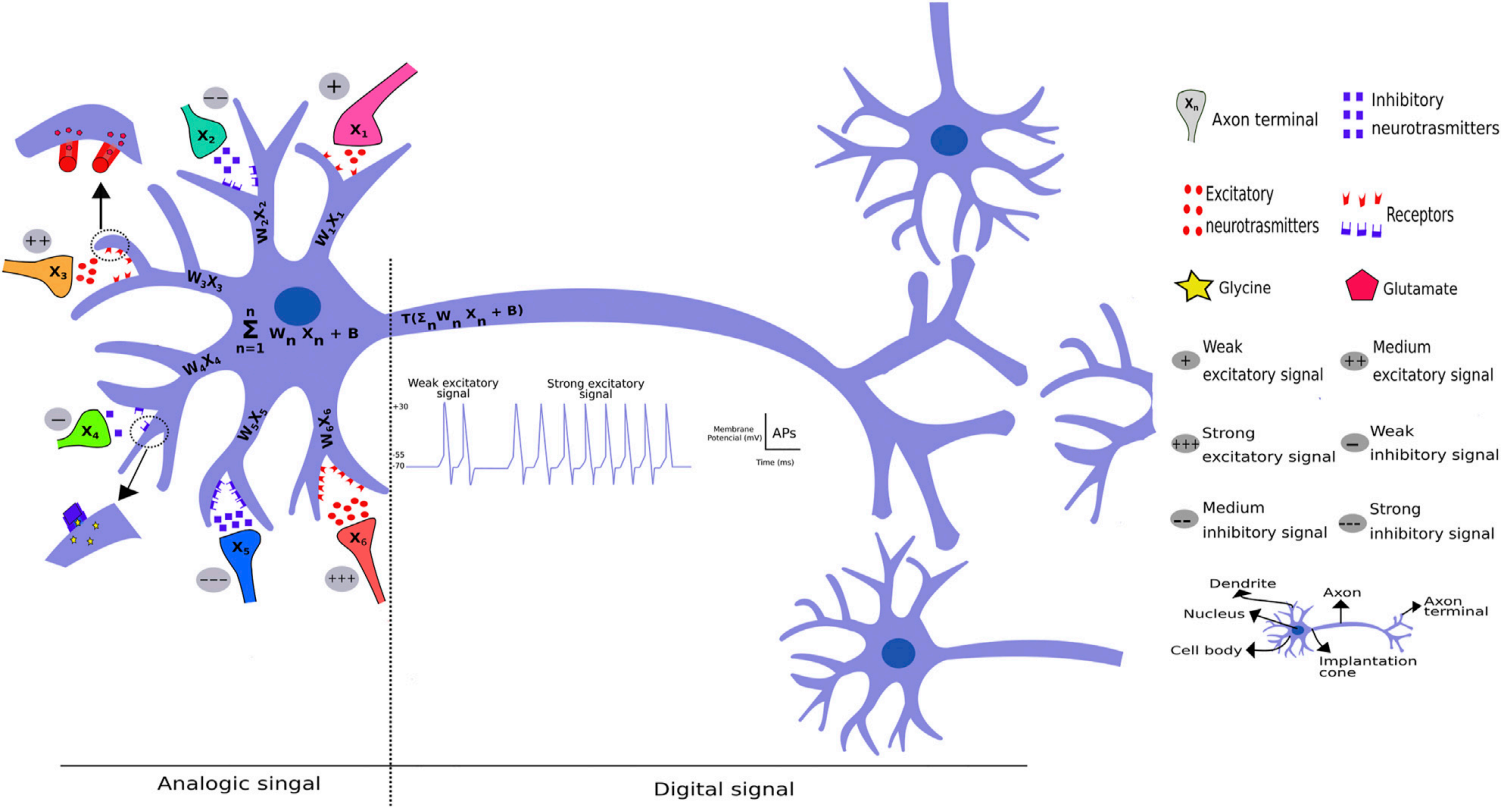
Comparative model of perceptron and real neuron. A neuron receives both excitatory and inhibitory signals through the release of different neurotransmitters from different biological neural networks that generates postsynaptic excitatory or inhibitory potentials. These potentials can add up with space or time in a continuous manner, thus classified as analogic signals. If this voltage reaches the potential threshold, it will trigger an AP in the axon implantation cone (hillock), which will lead to the passage of the signal through the axon and the axon terminals until it synapses with other neurons. This type of signal can be classified as digital, because it occurs (binary 1) or not occurs (binary 0), depending on the signal reaching the action potential threshold.

An interesting example comprises finger heat stimuli. When any hot object touches a human finger, it opens up the finger’s thermosensitive transient receptor potential (TRP) at the molecular level, non-selective cation channel (Lamas et al., 2019; Nadezhdin et al., 2021). This allows for cation entry, leading to membrane depolarization, i.e., receptor potential, an analogic response proportional to the thermal stimulus. If this stimulus reaches a threshold potential, it triggers an action potential (AP) comprising an all-or-none response (Adrian, 1914; Häusser, 2000) in the axon implantation cone, as depicted in Figure 2. The receptor potential, a type of an analogic signal, triggers an action potential that varies only in frequency, comprising a digital signal (Figure 3). A strong heat stimulus is differentiated from a weak one because, it increases action potential frequency, i.e., if the weak stimulus results in two AP per second, the strong one results in 10 per second. Thus, the signal presents a frequency code in most of animals studied to date. In the spinal cord, this signal releases an excitatory neurotransmitter in the synaptic cleft that will generate AP firing from an alpha motoneuron that innervates agonist muscles, thus, generating a withdrawal reflex (Figure 2). In addition, a synapse between the sensitive neuron with an interneuron that synapses onto, releasing an inhibitory neurotransmitter, an alpha motoneuron that, in turn, innervates antagonistic muscles, thus facilitating the withdrawal reflex, due to antagonistic muscle relaxation.

It is important to point out that the discovery of the action potential mechanism comprised a fantastic association of bench work and modeling performed by Huxley and Hodgkin with seminal papers published after the Second World War comprising a cornerstone of computational neuroscience (Schuetze, 1983; Hausser, 2020). In same period, McCulloch and Pitts (1943) were the first to mathematically model a neural network. Macculloch had an undergraduate degree in psychology and a graduate degree in medicine, unlike Huxley and Hodgkin, both engineers that worked on the Second World War radar defense system. Rosenblatt (1958), who held a PhD in psychology, and his team at the Cornell Aeronautical Laboratory developed the perceptron algorithm implemented in an IBM 704, consisting of the first “perceptron computer” to detect visual stimuli.

Several groups began studying neural network and learning employing perceptron techniques in the 1960s (Fradkov, 2020). This research, however, declined due to a book published in the 1970s, that demonstrated that this algorithm could only solve linearly separable problems, that kind of problems in which a graphical representation of the input stimuli would indicate two sets of stimuli, one associated with a “positive answer” and the other with a “negative” one (or none at all), allowing for the complete separation of these two sets by a straight line. Deep learning returned with a new framework introduced by Hilton’s team reintroducing the backpropagation algorithm (previously created by Bryson in 1963), findings lower cost functions through gradient descendent techniques (LeCun et al., 2015).

As depicted in Figure 3, a significant perceptron innovation comprised weights that act as excitatory and inhibitory neurotransmitters chemical synapses in neurons. The nervous system transduces external and internal signal as explained above through receptor potentials, excitatory postsynaptic potentials (EPSP), inhibitory postsynaptic potentials (IPSP), and action potential. The receptor potential and postsynaptic potentials can be added temporally and spatially, i.e., act as an aggregation function in perceptron and deep learning methods. These potentials can trigger an action potential in areas displaying a high number of voltage-gated sodium channels when the potential reaches a certain threshold. Thus, the analogic signal become a digital signal, comprising the action potential and, depending on the signal intensity, generates different frequencies to “pass along the message.”

In neural networks, all data must be transduced to numbers and transformed into a binary system to computer language. As depicted in Figure 1, representations of the external world or any phenomenon must be coded as a vector (1D tensor) or a tensor of any dimension. As described previously, this information is aggregated through a “summation” (S) function, as in the synaptic and receptor functions. The basic function is denoted as S = ∑ wx_n +_ b, with another representation (S = weight _*_ input signal + bias), where w represents adaptable weights, x are the numbers or number vector and b represents the bias, a term that avoids a zero signal and dislocates the curve in the y axes when working with only one variable. This S function containing one variable comprises a first degree equation well studied in elementary school. The next function is an activation function, a mathematical representation of a “set of sodium gated voltage channels.” This function (herein named T) triggers a response, through different functions, similar to the action potential that occurs in the hillock, a region that connects the cellular neuron body to the axon, in reach of the sodium voltage-dependent channels (Figure 3).

**FIGURE 4:**
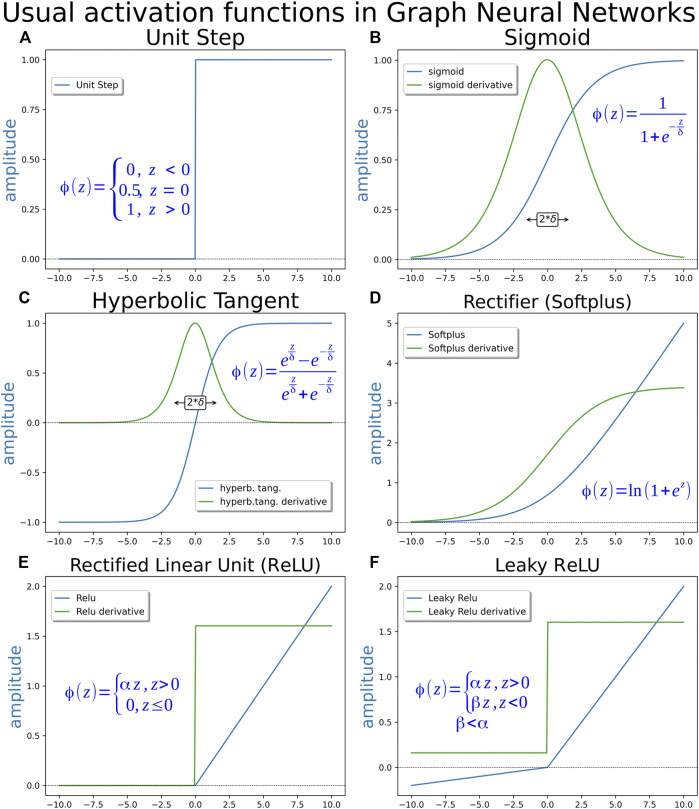
Activation functions. **(A)** Unity step function, binary step function or heaviside function–This function was used in the early perceptron era. It is unsuitable for gradient descent learning methods, since its derivative is 0 along almost the entire domain. **(B)** Logistic or sigmoid function—This is perhaps the most extensively applied activation function. Its answer may become steep, depending on the value of parameter δ. **(C)** Hyperbolic tangent (TanH)—This function is used as an alternative to the sigmoid function when a range of output from −1 to 1 is needed, instead of the range from 0 to 1 given by the sigmoid function. **(D)** Softplus function or rectifier (as the shape of the function resembles the behavior of a rectifier diode)—It is differentiable along the entire domain as the two previous functions, but displays an advantage, since its derivative exhibits non-zero values along the entire positive domain while sigmoid and hyperbolic tangent present derivatives approaching zero asymptotically for large argument values. A non-zero derivative along a broader range may contribute to speed up the learning process. The derivative of the softplus function is the sigmoid function. **(E)** Rectified linear unity function (ReLU)—This function is used as a simple linear alternative to the softplus, maintaining the same advantage of a non-zero derivative along the entire positive domain but suffering the problem of a discontinuity in the derivative for argument equal to 0. **(F)** Leaky ReLU—This function is a “leaky” form of ReLU. The answer of this function in the negative domain comprises an attenuated version of the linear response in positive domain, instead of 0. Depending on the attenuation parameter being a constant or an adjustable value, the function may be also named Parametric ReLU. The non-zero negative domain provides some decrease in the occurrence of dead neurons, the region of ReLU curve where any signal corresponds to zero as a response, in the network.

The first applied T function was a step function where, if the signal were more than or equal to zero, the response would be 1 otherwise and, if not, the response would be zero (Figure 4A). The binary step function can identify only two classes and work well as a first proof of concept in MacCulloch and Pitts´s Perceptron. Thus, several other activation functions were introduced to deal, with no linear events such as sigmoid, hyperbolic tangent (TanH), rectified linear unit (ReLU), Softplus, among others (Figures 4B–F). They are used as non-linear modulating elements in neuron output generation, resembling a decision-making process. Since the beginning of neural network sciences, with the perceptron, neural networks are used as a means to obtain a binary answer from linear data inputs. Learning techniques in neural networks are mostly based on gradient descent techniques, so, being differentiated along the entire range is a desirable quality for an activation function. Some examples of most used activation functions are displayed in Figure 4. Perceptron comprised the first “mathematical” neuron model created by researchers in the psychology field based on a real neuron. Deep neural networks and their consequent AI have evolved in a different direction that is not necessarily equal to a mammalian brain. Currently, computer neuroscience is dealing with “real” brain modeling processes, although cross talking is now implemented to understand the brain function (Fan and Markram, 2019; Savage, 2019).

### Graph Neural Networks

**FIGURE 6:**
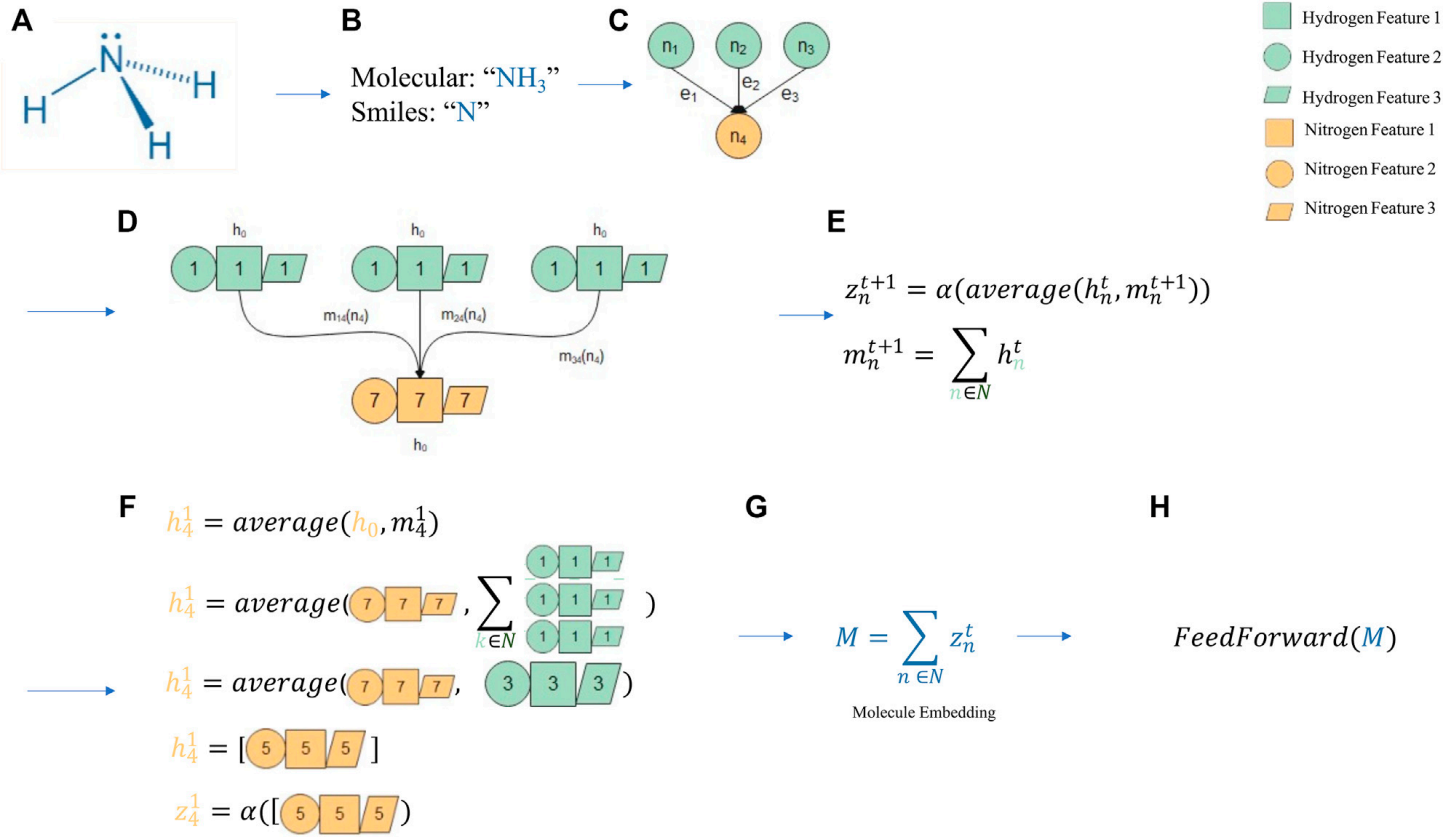
An intuition of a Graph Neural Network. A molecular prediction process scheme employing a message passing neural network (MPNN) as molecule embedder. **(A)** Ammonia was chosen as a candidate for prediction. **(B)** Text representation of ammonia as a simplified molecular-input line-entry system (SMILES) sequence and molecular formula. **(C)** The SMILES sequences are converted to a graph with three edges representing a molecular bond between hydrogen (green) and nitrogen (yellow). **(D)** h0 is the feature space for a specific atom, where each feature, herein represented as a geometric form, is didactically associated with the corresponding atom number. **(E)** These features comprise the atom features, which are averaged with the message function, herein as a sum, in each step of the MPNN training. **(F)** An example of how the first step of the MPNN is computed for nitrogen (n4) is provided. **(G)** The final step of MPNN is a feature embedding for each atom, which can be summed to be then used by a feed-forward network for final predictions. **(H)** M is the feature embedding for each atom, consisting in a shape of (N Atoms x E Embedding space). This (NxE) tensor can be feeded into a more simplistic network architechture, i.e.: Feed Forward to train task-specific models.

Graph theory has been part of mathematics curriculum since 1736, due to Euler’s seminal work and has been incorporated in computer science in the modern age, but there is evidence of rudiments of graph theory before Euler’s study (Euler, 1736, Alexanderson, 2006). In the two-dimensional plane, the graph is constituted by set of vertices (V), also named nodes, and edges (E) that link the vertices. The general mathematical representation of this graph is G = (V, E). Graphs are a quite general kind of data representation that can model relationships of almost everything, from galaxies to subatomic particles. Social networks, roads linking cities, brain connections, protein and DNA interactions, among others, are examples of relationships that can be modeled as graph structures. Several subtypes of graph neural networks have been developed, as depicted in Table 1. Despite several a particularity, all have a similar workflow, as displayed in Figure 6, where an illustrative example of a prediction using message passing neural network (MPNN) is provided. In general, a molecule can have many representations, including graphical (such as 3D structures), textual (i.e., molecular formula), and others. One way of representing a molecule is to use graphs that work as a kind of an abstract version of a molecular modeling kit. The instructions to mount a given molecule model with N atoms labeled by sequential numbering (formally known as nodes) can be given by a table with N rows and N columns, where the existence of a bond (named edge) between each pair of atoms *i* and *j* is represented by a value 1 in row *i* and column *j* in the given table (named adjacency matrix), and with every other position in the table presenting value 0.

**TABLE 1:** Summary of main graph neural network architectures.

GNN	Main characteristics	Examples	Applications	References
Graph Convolutional Networks (GCN)	GPU optimization is possible	Diffusion Convolution Neural Networks	Protein interface prediction	Zhou et al. (2020)
	Avoid vanishing gradients	GraphWaveNet	Image classification	Wu et al. (2021)
	Allow neighborhood-level aggregations		Social networks	
Graph Attention Networks (GAN)	Quadratic complexity	Graph Attention Network	Molecular feature prediction	Velicković et al. (2018)
	Useful to model positional-dependent data in nodes		Traffic modelling	Zhou et al. (2020)
			Image classification	
Graph Recurrent Networks (GRN)	May not be GPU-optimizable	Gated Graph Recurrent Networks	Epidemic progression modelling	Ruiz et al. (2020)
	Allow for modelling the graph structure over time	Graph Recurrent Network	Time series	Wu et al. (2021)
	Can approximate any Borel function			
Message Passing Neural Networks (MPNN)	Locally derived embeddings	Chemprop	Drug repurposing	Zhou et al. (2020)
	Allow for edge and nodes feature inputs	Directional Message Passing Networks	Molecular modeling	Wu et al. (2021)
	Universal approximators are not good	Bayesian Graph Neural Networks		
Graph Autoencoder (GAE)	Allow for graph generative networks	MolGAN	Drug discovery	Wu et al. (2021)
	Latent space projections	NetGAN	3D modelling	
			Dimensionality Reduction	

**FIGURE 5:**
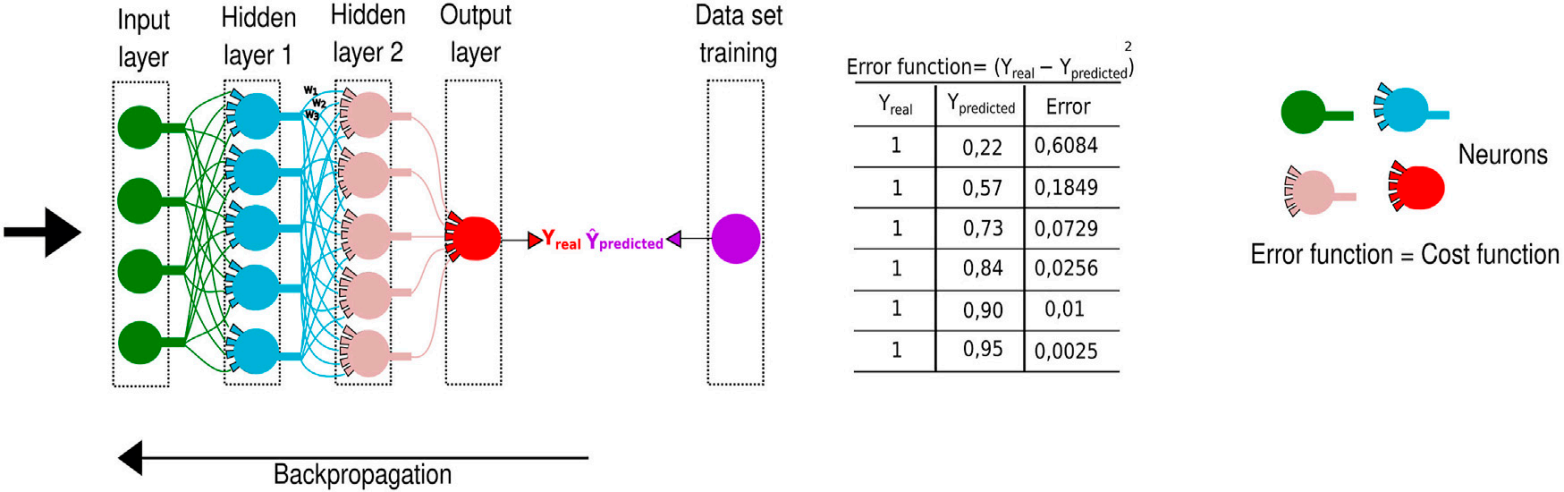
Representation of a deep neural network. It is widely applied in computing systems, employing schematic neurons to demonstrate the signal passage through different layers. The predicted value named ŷ is obtained in the final layer. This value will be decreased from the real y value, and the result will comprise the error, of what was lost along the neural network. The process is repeated until the smallest possible error is reached through a backpropagation algorithm.

It is possible to extrapolate a molecule representation in the same graph-like structure of a social network, where atoms comprise the people and bonds are equivalent to relationships. It is also possible to convert a textual representation into a graph representation. Figure 6 provides an example of this conversion with ammonia, represented as its molecular formula and SMILES (simplified molecular-input line-entry system). The latter presents some conventions that make molecule interpretation easier by computer programs. The sequence is converted into a graph displaying the aforementioned edge and bond relationship. Interestingly, graphs allow for not only modeling relationships, but also for adding node and edge information. In the parallel of a social network, it is possible not only to store how friends are related, but the name of each friend (which would be node-added information) or when the friendship began (edge-added information). In a molecular context, this allows for information on atoms (i.e., hybridization) and bonds (i.e., bond type) to be stored alongside the bonds and atoms that represent the molecule itself.

Usually, information coding concerning atomic features corresponding to one value choice among a set (for example, the kind of the atom in a node being chosen from a list of M possible atoms) may be performed by “one-hot” coding, where the position in the list that corresponds to the specific feature of the atom occupying the node being set to 1 and the remaining M-1 positions, to 0. For example, considering a short list of possible atoms as (H, C, N, O, F, S, Cl, Other), the nitrogen in the ammonia molecule would be represented by (0,0,1,0,0,0,0,0) and each of the hydrogen atoms by (1,0,0,0,0,0,0,0). Features that can be expressed by integer values may be coded by the integer value itself or also by “one-hot” coding considering a list of all possible values attributable to the investigated feature. For each atom, considering an entire set of atomic features is obtained by concatenating the codes attributed to all these features in the form of a vector in a defined order.

Depending on the set of features, it is usual for these vectors to present a dimension around one hundred or more. The features of the entire molecule are thus, represented by a matrix whose rows corresponds to the feature vectors of the atoms according to the sequential order in which they are labeled. As result, even small molecules may be represented by matrices with hundreds to thousands of values. A neural network layer with a matrix of features like these as input and a number of neurons about the same order as outputs would easily surpass millions of parameters to be adjusted by training.

**FIGURE 7:**
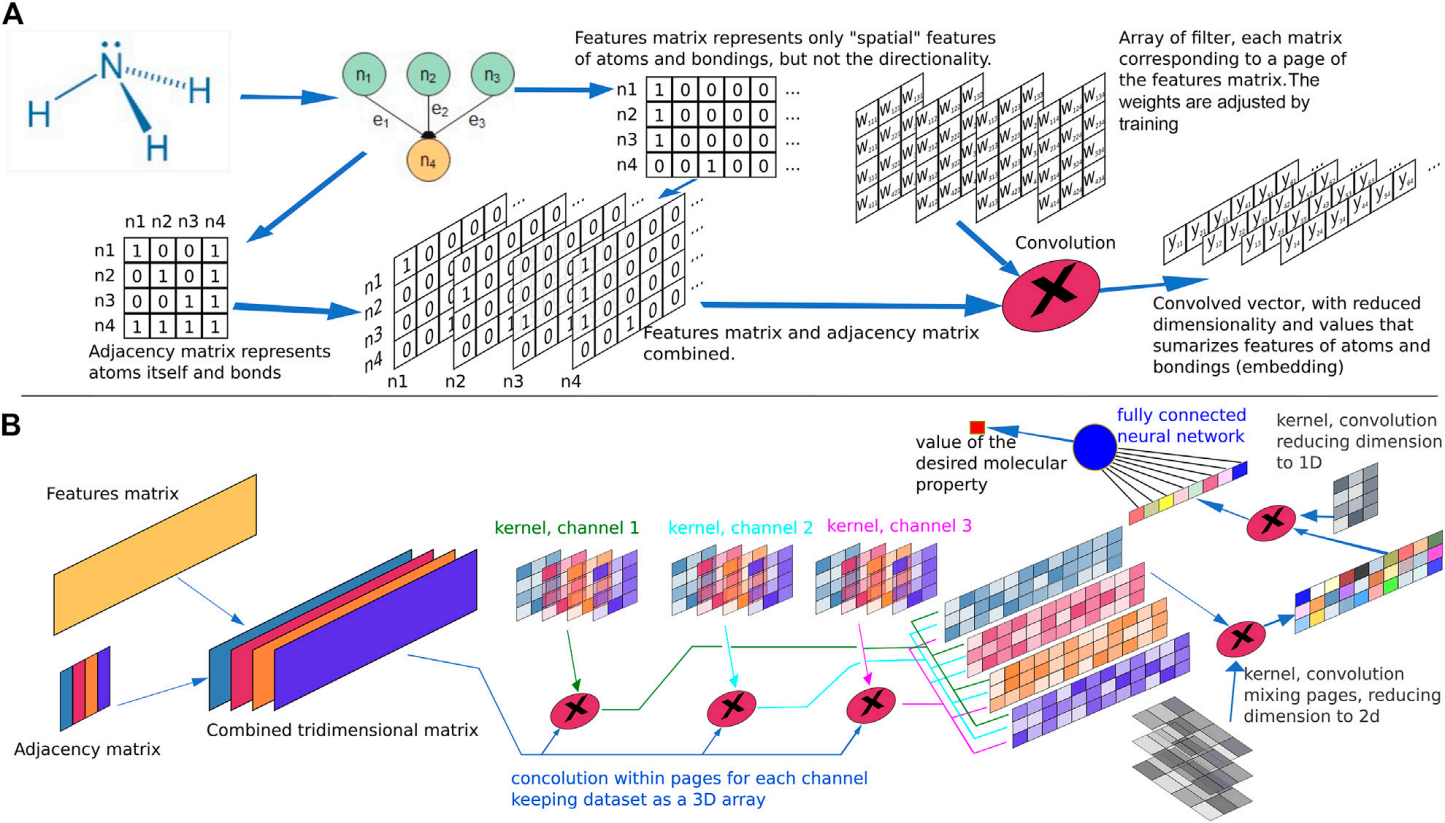
Molecular property representation. **(A)** Example of an atomic feature matrix and an adjacency matrix for ammonia. The elements i, j in the adjacency matrix are representations of the connection between atoms i and j. A combination of the feature and adjacency matrix is performed by column wise multiplication, resulting in a 3D array. Each atomic bond corresponds to a “page” in the array. A filter (kernel) corresponding to a 3D matrix with the same number of pages as the combined data set feature-adjacency is applied to the matrix through a scalar product. The filter elements, when inserted in a neural network comprise weight parameters for the connections and its values are adjusted by training, defining the filter characteristics in an optimized manner. The offset of the filter with respect to the data set is swept to cover the entire indices range. The result of the convolution operation in this example is a 2D array with four rows and N-2 columns, where N is the number of columns in the feature matrix. **(B)** Example of a hypothetical neural network used to calculate the value of a property of a given molecule. Several convolution layers are chained to perform the embedding of the data representing the atomic features and the atomic bonding, the result being a 1D vector which is further submitted to a fully connected neural network. The output of this network is the desired parameter. The training algorithm adjusts the parameters of all kernel filters and the weights of the output neural network, until the error of the predictions compared to the training set are minimized.

In this context, convolution techniques have been proven useful to enhance molecular features while, at the same time, significantly reduce the dimension of the feature matrix, as depicted in Figure 7. Convolution has been extensively employed in image or language processing and consists in multiplying a small matrix, known as “filter” or “kernel,” by the data matrix (the feature matrix, for example). The dimension of the filter usually comprises few rows and columns and the multiplication takes the same number of elements as the filter from the data matrix, considering the element in the first row and the filter column being aligned with one element in the data matrix in a variable position. Thus, for each element in the filter a corresponding element is noted in the data matrix with an equivalent offset of rows and columns regarding the first filter element. The data matrix and filter elements are multiplied position by position and summed (an operation equivalent to the scalar product). To cover the entire data matrix, the scalar product is obtained for each alignment position by moving the filter from the first row and first column of the data matrix, step by step, and the amplitude of the step, known as “stride,” corresponds to a value in the range from 1 to the corresponding filter dimension.

Comparing the dimension of the original data matrix, of N_0_ rows by D_0_ columns, with to the corresponding dimension of the convoluted matrix, with N_1_ rows by D_1_ columns, and convolution filter displaying a dimension J rows by K columns and a stride value of S_r_ and S_c_ for rows and columns, respectively, the following relationship arise:

$${\text{N}}_{1}=\left({\text{N}}_{\text{0}}-\text{J}\right)/{\text{S}}_{\text{r}}+1\text{;}\;{\text{D}}_{1}=\left({\text{D}}_{\text{0}}-\text{K}\right)/{\text{S}}_{\text{c}}+1;$$

Sometimes, when dimensionality reduction is not a desired convolution result, a convenient filling with all zero to rows and columns around the data matrix may be performed previously to convolution, known as “padding.” Considering the addition of P_r_ rows with zeros above and below the data matrix and P_c_ columns with zeros on the left and right of the data matrix, the relationship above becomes:

$${\text{N}}_{1}=\left({\text{N}}_{\text{0}}{+2\text{*P}}_{\text{r}}-\text{J}\right)/{\text{S}}_{\text{r}}+1\text{;}\;{\text{D}}_{1}=\left({\text{D}}_{\text{0}}{+2\text{*P}}_{\text{c}}-\text{K}\right)/{\text{S}}_{\text{c}}+1\text{;}$$

Additionally, techniques combining the features matrix with the adjacency matrix forming a multidimensional array followed by use of convolution techniques to obtain smaller arrays or even vectors as a result, a general process usually known as “embedding,” provides a more compact representation joining atomic and bond features. This representation is much more convenient to be used as input in a final fully connected feed forward neural network to obtain the final value of the molecular property of interest being modeled, such as binding energy, affinity score or similar.

Chaining groups of graph representation layers and graph convolution layers in a network structure to predict molecular properties has been reported as achieving superior performance in some molecular property predictions (Wang et al., 2019).

Storing information on each atom and bond provides a convenient and generalized way of passing information forward in a local perspective. For example, analyzing the Fab region of an immunoglobulin may be much more informative than analyzing its Fc region when designing a vaccine. However, the data representation is not significant without a model that leverages its advantages. In this example, MPNN is used. MPNNs are interesting in contrast to other kinds of GNNs, as they can model how node and edge information relate in a local context (Figure 6). For example, when a hydrogen is added to ammonia, it becomes ammonium. Despite having the same single bond to the new hydrogen as it had to the previous hydrogen atom, ammonium displays many different properties compared to ammonia. The addition of a new hydrogen affects the other atoms in the molecule, altering its shape from triangular pyramidal to tetrahedral. This can be construed as the message propagating from one atom and bond (from the nitrogen to the new hydrogen) to the others atoms (the remaining hydrogens). This message function is one of the learned functions within the MPNN. In the end, the purpose of the MPNN is to convert the unstructured data of a graph (which previously comprised a simple text) into a semantic embedding which essentially comprises a tensor assumed to be the best molecule summarization. This summarization in the form of a tensor can be applied to any subsequent task, such as predicting blood-brain barrier permeability. The base assumption is that learning how to best summarize the molecule should simplify any following prediction that employs this summarization, allowing for less data to be used (i.e., few-shot learning).

### GNN Applications to VS

Recently, Wieder et al. performed a literature survey accounting for about 80 different GNN models in 63 publications, which were applied to different fields such as quantum chemistry, physicochemical property predictions, biophysics, biological effects, and synthetic accessibility (Wieder et al., 2020). This section discussed some recent GNN applications to VS field.

Currently, an increasing number of articles describing new frameworks to predict interactions between ligands and proteins is noted (Jin et al., 2021). Interestingly, these graph-based neural networks are gaining new adaptations and, because of this, constantly exhibit better performance than conventional molecular docking programs, such as Autodock Vina.

In this regard, Torng and Altman implemented a framework using graph convolutional neural networks (GCNN) to predict protein-ligand interactions among 102 protein targets. This model demonstrated better performance of 0.886 area under curve (AUC) when compared to other programs, such as 3DCNN (0.868), Autodock Vina (0.716), RF-score (0.622), and NNScore (0.584) (Torng and Altman, 2019). Lim et al. also proposed a novel approach to perform structure-based VS using GNN based on the 3D protein-ligand binding pose. This model was superior to the Torng model (0.968 vs. 0.886) and both CNN and docking (0.968 vs. 0.868 and 0.689, respectively), besides having presented a balanced accuracy of 90.9%. However, when the ChEMBL database was employed, the values of area under curve-receiver operating characteristic (AUROC), sensitivity, specificity, and balanced accuracy significantly decreased (Lim et al., 2019).

Jiang et al. created an accurate model (<96%) to predict drug-target interactions, based on the construction of two graphs: one for the molecule according to its SMILES sequence, and one protein graph built from a contact map of the protein sequence. Subsequently, two GNN extracted the information and were able to predict the affinity of the ligand and the target protein (Jiang et al., 2020).

Furthermore, GNN algorithms can be used to predict EC_50_, solubility, and molecular properties. They are also able to perform molecular dynamics simulations (Duvenaud et al., 2015; Klicpera et al., 2020).

Although GNN show better results in terms of accuracy than molecular docking methodologies, for example, their applications to VS are still scarce and studies are still recent. This demonstrates that VS is still not able to keep up with the growth rate of the improved GNN models currently being produced. However, this will probably change soon. VS results obtained by GNN are summarized in Table 2.

**TABLE 2:** Recent GNN applications in VS.

Model	Application	Number of molecules tested	Libraries	References
Graph convolution network (GCN)	Drug repurposing	>3,000	RepoDB	Wang et al. (2020)
Graph neural network (GNN)	Drug repurposing	3,635	CTDbase’s COVID-19 curated list	Hsieh et al. (2020)
Graph neural network (GNN)	Drug discovery	>10,000	ChEMBL and ZINC	Zhi et al. (2021)
Directed message passing neural network (D-MPNN)	Drug discovery	>107 million	Drug Repurposing Hub and ZINC15	Stokes et al. (2020)
Pre-trained self-attentive message passing neural network (P-SAMPNN)	Drug discovery	792	SPECS natural products	Liu et al. (2021)

A promising GNN application in VS concerns drug repurposing. Wang et al., for example, proposed a bipartite graph convolution network model for drug repurposing that outperformed conventional GCN (0.857 vs. 0.792). In addition, among the top five rank-predicted list for breast carcinoma, four molecules were validated by the literature, namely clofarabine, cimetidine, thiamine, and arsenic trioxide, which present approximately 80% success rate. For Parkinson’s disease, five drugs among the top ten predicted drugs presented literature validation. They are dextromethorphan, solifenacin, atomoxetine, venlafaxine, and tapentadol (Wang et al., 2020).

In another study, Hsieh et al. used GNN methodology to discover repurposable drugs to treat COVID-19. Their model was constructed based on the SARS-CoV-2 knowledge graph map, which considers several virus interactions such as baits, host genes, pathways, phenotypes, and drugs. Their work highlighted 22 potential drugs (Hsieh et al., 2020).

Recently, Zhi et al. performed an interesting screening work to discover new dihydroorotate dehydrogenase protein inhibitors, considered an important molecular target for the treatment of small cell lung cancer. After gathering information from molecular docking, GNN and ML algorithms, the authors found three molecules with the desired activity: folic acid (ZINC8577218), thioguanosine 5′-triphosphate (ZINC95618747), and ATP (ZINC4261765). They also performed molecular dynamics simulations to confirm the interaction between these molecules and the aforementioned protein (Zhi et al., 2021).

Stokes et al. used a directed-message passing neural network (D-MPNN) to screen molecules displaying antibiotic activity. They reported that halicin, a c-Jun N-terminal kinase inhibitor, significantly reduced bacterial growth both *in vitro* and *in vivo* through dissipation of bacterial transmembrane ∆pH potential. Halicin also demonstrated activity against several bacterial strains, including *Escherichia coli*, *Mycobacterium tuberculosis*, *Acinetobacter baumannii*, and *Clostridium difficile*. Moreover, another two molecules (ZINC000225434673 and ZINC000100032716) significantly inhibited *E. coli* growth *in vitro*, and similarly to halicin, displayed a distinct structure from conventional antibiotics (Stokes et al., 2020).

Liu et al. performed a VS to discover novel anti-osteoporosis drugs from natural products using a pre-trained self-attentive message passing neural network (P-SAMPNN). Among the five hits selected for *in vitro* tests, a laudanosine derivative and a codamine derivative exhibited activity at the nanomolar range (i.e., 32 and 68 nM, respectively), suppressing osteoclastogenesis-related genes (Liu et al., 2021).

### Graph Neural Networks Performance Evaluations

Depending on the problem, both graph-based networks and traditional descriptor-based networks may be used for regression or classification tasks. Results quality assessments depend on the definition of a suitable metric parameter. In case of regression tasks, root mean square error (RMSE) or determination coefficient (R^2^) are the most employed metric parameters. Classification tasks, accuracy, logarithmic loss, area under receiver operating curve (AU-ROC), area under precision recall curve (AU-PRC), among others, are the most frequent metrics. For a description of these and others, detailed descriptions are available elsewhere (Liu et al., 2014).

Model comparisons also depend on the choice of suitable metrics that may be calculated for these models. The set of models is tested by making predictions for each one when applied to processing data from a common set of databases. Several public and proprietary databases are available. Among public databases, the most frequently employed are: ESOL, a water solubility database for organic small molecules; FreeSolv, a database for hydration free energy database for small molecules in water; Lipop, a logarithmic octanol/water distribution coefficient database at pH = 7.4; MUV, a subset of PubChem BioAssay by applying a refined nearest neighbor analysis, designed for the validation of VS techniques; HIV, containing data concerning HIV replication inhibition; BACE, containing data on the inhibition of human β-secretase; BBBP, containing binary blood–brain barrier penetration; labels Tox21, ToxCast, SIDER, and ClinTox, containing data related to toxicity measurements or qualitative evaluations for many biological subjects and including data about on drug toxicity and side effects in clinical trials; and CheMBL, a chemical database for bioactive molecules with drug-like properties, among others. All these datasets are availabe in the MoleculeNet benchmark framework (Wu et al., 2018).

Extensive literature reports concerning the benchmarks of algorithm models using the aforementioned databases applied to VS related tasks are available, such as molecular property predictions, fingerprint generation or the evaluation of structural protein-ligand docking parameters. These include the following: Support Vector Machine (SVM), Extreme Gradient Boost (XGBoost), Random Forest (RF), and Deep Neural Networks (DNN) (Jiang et al., 2021) as representatives of descriptor-based models and many graph-based algorithm variants, such as MPNN—Message Passing Neural Networks (Yang et al., 2019; Deng et al., 2021; Jiang et al., 2021) and networks implementing algorithm model variants involving spatial graph convolution, like GCN—Graph Convolution Network (Li et al., 2017; Xiong et al., 2020; Menke and Koch, 2020; Deng et al., 2021; Hsieh et al., 2020) or GC—Graph Convolution (Wu et al., 2018) and spectral graph convolution, such as AGCN–Adaptive Graph Convolution (Li et al., 2018), graph based networks including attention mechanisms of interaction between near nodes or edges, i.e., AFP—Attentive Fingerprint (Xiong et al., 2020; Jiang et al., 2021), PAGTN—Path-Augmented Graph Transformer Network (Chen et al., 2019), EAGCN—Edge Attention GCN (Shang et al., 2018), among others (Wu et al., 2018; Lim et al., 2019).

Best performance values are mostly associated with graph-based models, with few exceptions comprising non-graph models performing better than graph models when applied to specific databases and properties (Jiang et al., 2021), with SVM, RF and XGBoost providing the best AUC-ROC values.

There may be enough room to find synergism in combinations of graph based on descriptor based models to achieve improved results.

Direct Message Passage Neural Network, D-MPNN—a graph based model combined with Extreme Gradient Boost, XGBoost—a descriptor-based network as the output layer, achieved the best results for several of the presented dataset (Deng et al., 2021). Furthermore, the concatenation of molecular fingerprint vectors generated by conventional models with descriptors generated using graph models have been reported as providing the best prediction results when submitted to the final parameter generation layers (Wang et al., 2019).

### Graph Neural Network Limitations

Despite being applicable to a handful of fields that could benefit from ML techniques, GNNs also have drawbacks and limitations. First, while conventional ML techniques such as Logistic Regressions usually display a limitation regarding the ability to leverage the information gain with increasing dataset sizes, DL techniques display the increased benefit of employing huge datasets. However, DL methods also tend to underperform under small data environments (Thompson et al., 2020). Secondly, GNNs are also more prone to suffer from small data perturbations (Zügner and Günnemann, 2019), becoming vulnerable to adversarial attacks. Moreover, some types of GNNs are not injective. Exemplifying, Graph Convolutional Networks (GCNs) use mean pooling to aggregate node multisets, being not injective due to this mean aggregation, GCNs are not able to distinguish nodes that receive messages from N nodes and nodes that receive messages from M > N nodes. The limitation regarding being non-injective is also shared with other GNN architectures, such as GraphSAGE, yet this is solved in MPNNs. Furthermore, many types of GNNs (i.e.,: GCN, GraphSAGE, GIN and GAT) that rely on local information are not able to compute important graph properties in simple graphs, such as clique information, longest or shortest cycle and diameter (Garg et al., 2020).

Graph-based models tend, however, to demand much higher computing time than descriptor-based models (Jiang et al., 2021). Descriptor-based models may therefore still be, in some cases, a compromise between acceptable performance results and shorter computing times. In general, graph-based methods tend to present higher computational costs and yield less promising predictions than descriptor-based methods (Jiang et al., 2021). Yet, when evaluated on 127 ChemBL diverse targets, GNNs present high predictability (Sakai et al., 2021). Dwivedi et al. (2020) provide a comprehensive benchmark between GNNs and other ML methods, stating that GNNs outperform other ML methods such as Multilayer Perceptrons for chemical predictions. Yet, regarding SMILES sequences being similar to Natural Language Processing (NLP) sequences, further assessments on evaluating GNNs against Transformer-based DL methods, that could simplify inputs and are better described in NLP applications, are required.

## Conclusion and Future Perspectives

The studies pointed out in this paper indicate that the use of GNN in VS may guide the drug discovery process. However, the application of this model in the field of natural products is still underexplored. Nevertheless, several already interesting tools produced with deep learning algorithms have been developed that can aid in the drug discovery process involving natural products. The NPClassifier developed by Kim et al., for example, can aid in the recognition of the structural diversity of organisms such as fungi and bacteria, in addition to discovering which organism produces a particular chemical class that has displayed VS activity in VS campaign (Kim et al., 2020). In another assessment, Roberts et al. employed a deep convolutional Siamese network to map 2D Nuclear Magnetic Resonance (NMR), which helps determine the structure of novel compounds (Roberts et al., 2019). Recently, Yoo et al. developed a deep learning approach able to extract the molecular and chemical properties from natural products and predict their medicinal use for the treatment of several diseases, including hypertension, pain, diabetes mellitus type 2 and rheumatoid arthritis, among others. This approach was also developed to accelerate SV campaigns, as it allows for a preliminary screening to identify molecules with potential activity from a vast database (Yoo et al., 2020). Reher et al. also developed a novel NMR-based ML approach named SMART 2.0 (Small Molecule Accurate Recognition Technology), that identifies major constituents from crude extracts and was responsible for the identification of several compounds from cyanobacterial extracts, including symplocolide A, swinholide A, and samholides A-I (Reher et al., 2020). In sum, these findings indicate that the use of deep learning tools can aid in overcome the long-standing challenges surrounding natural product research, as well as accelerate the drug discovery process.
